# Neutrophil Elastase-Generated Fragment of Vascular Endothelial Growth Factor-A Stimulates Macrophage and Endothelial Progenitor Cell Migration

**DOI:** 10.1371/journal.pone.0145115

**Published:** 2015-12-16

**Authors:** Elma Kurtagic, Celeste B. Rich, Jo Ann Buczek-Thomas, Matthew A. Nugent

**Affiliations:** 1 Department of Biochemistry Boston University School of Medicine, Boston, Massachusetts, United States of America; 2 Department of Biological Sciences, University of Massachusetts Lowell, Lowell, Massachusetts, United States of America; Centro Cardiologico Monzino, ITALY

## Abstract

Elastase released from neutrophils as part of the innate immune system has been implicated in chronic diseases such as emphysema and cardiovascular disease. We have previously shown that neutrophil elastase targets vascular endothelial growth factor-A (VEGF) for partial degradation to generate a fragment of VEGF (VEGFf) that has distinct activities. Namely, VEGFf binds to VEGF receptor 1 but not to VEGF receptor 2 and shows altered signaling compared to intact VEGF. In the present study we investigated the chemotactic function of VEGF and VEGFf released from cells by neutrophil elastase. We found that endothelial cells migrated in response to intact VEGF but not VEGFf whereas RAW 264.7 macrophages/monocytes and embryonic endothelial progenitor cells were stimulated to migrate by either VEGF or VEGFf. To investigate the role of elastase-mediated release of VEGF from cells/extracellular matrices, a co-culture system was established. High or low VEGF producing cells were co-cultured with macrophages, endothelial or endothelial progenitor cells and treated with neutrophil elastase. Elastase treatment stimulated macrophage and endothelial progenitor cell migration with the response being greater with the high VEGF expressing cells. However, elastase treatment led to decreased endothelial cell migration due to VEGF cleavage to VEGF fragment. These findings suggest that the tissue response to NE-mediated injury might involve the generation of diffusible VEGF fragments that stimulate inflammatory cell recruitment.

## Introduction

The development and progression of pulmonary emphysema is characterized by tissue destruction, uncontrolled elastase activity, alveolar apoptosis, reduced alveolar capillary density and altered extracellular matrix (ECM) mechanics [1–5]. Vascular endothelial growth factor-A (herein referred to as, VEGF) is critical for maintenance of the pulmonary capillary bed, with increased or decreased VEGF being associated with disease [6–9]. Specifically, reduced VEGF and VEGF receptor 2 (VEGFR2) and endothelial cell apoptosis have been linked to the tissue destruction associated with pulmonary emphysema [10–13]. Thus, vascular dysfunction is a crucial component of the development and progression of emphysema, with VEGF being central to this process.

We have previously found that VEGF is a substrate for neutrophil elastase (NE) cleavage leading to the generation of a VEGF fragment (VEGFf) that shows altered activity. Namely, it binds VEGFR1 and has lost the ability to bind to VEGFR2, the VEGF co-receptor, neuropilin-1 (Nrp1), and fibronectin and heparan sulfate in the ECM [14, 15]. Mass spectrometry analysis of VEGFf shows that NE cleaves the N- and C-termini as well as internal regions that likely lead to loss of the structural motif involved in VEGFR2 binding [15]. NE has been implicated in the generation of emphysema and has been shown to participate in pathologies such as arthritis, aneurysms, atherosclerosis and other chronic conditions related to alterations in structural tissues. In all these diseases there is a significant vascular component associated with endothelial cell dysfunction. VEGF is a critical factor for endothelial cell survival in various tissues including but not limited to pulmonary and vascular systems. Interestingly, VEGF has been considered a potent promoter of vascular and myocardial repair [16–18]. Therefore, it is possible that NE and VEGF may interact to play roles in chronic disorders, where proteolytic degradation of the ECM by NE might impact VEGF storage and release. For instance, VEGF release from extracellular matrices might regulate inflammatory and progenitor cell recruitment and activity, modulating inflammatory response and potentially mediating tissue repair.

NE is also known to modulate the activation of platelets, promoting aggregation and augmenting both thrombosis and fibrinolysis by cleavage of clothing factors and their inhibitors [19]. NE has also been implicated in vascular plaque development [20, 21] where a subpopulation of plaque macrophages appear to express NE that participates in cytokine activation and the consequent migration of macrophages, influencing plaque stability. These findings suggest that excessive proteolysis by unregulated NE may play a broad role in modulating inflammatory processes through mechanisms that are independent of its ability to degrade elastin.

There are few studies evaluating the direct relationship between NE and VEGF. An interesting potential link between VEGF and the classic elastase:antielastase hypothesis is that VEGF is stored within the ECM. Thus, elastase injury to the ECM is likely to have an impact on storage, release, and activity of VEGF. We investigated the potential link between NE-mediated injury and the VEGF pathway. We show the NE-injury of VEGF-rich matrices leads to enhanced migration of RAW264.7 macrophages and embryonic endothelial progenitor cells (eEPCs) through the action of VEGFf. These findings suggest a new mechanism where the repair of tissue injured by excessive elastolysis might involve not only nascent activities of growth factors but also new activities generated by direct cleavage of growth factors during the tissue injury process.

## Materials and Methods

### Materials

Human recombinant VEGF-A_165_ produced in *Spodoptera frugiperda* cells from R&D Systems (Minneapolis, MN) was used for all experiments. VEGF is highly conserved across all species used in this study (bovine, rat, mouse) with no known distinctions in activity in cells from these species [22, 23]. Recombinant human TNF-α, Quantikine human VEGF immunoassay enzyme-linked immunosorbent assays (ELISAs), recombinant human Fc-VEGFR2 chimera and Fc-VEGFR1 chimera were purchased from R&D systems (Minneapolis, MN). Bovine serum albumin (BSA) was obtained from American Bioanalytical (Natick, MA). RPMI-1640 low glucose media was purchased from Lonza Walkersville (Walkersville, MD). Dulbecco’s Modified Eagle’s media (DMEM), phosphate buffered saline (PBS) containing no Ca^2+^ and Mg^2+^, penicillin/streptomycin, L-glutamine, N-(2-hydroxyethyl) piperazine-N’-2-ethanesulfonic acid (HEPES) and trypsin-EDTA were obtained from Invitrogen (Carlsbad, CA). Calf serum (CS) and fetal bovine serum (FBS) were purchased from Hyclone (Logan, UT). Human NE and VEGFR kinase inhibitor III (SU5416) were purchased from Calbiochem (San Diego, CA). NE was obtained from Elastin Products (Owensville, MO).

### Preparation of VEGFf

VEGFf was prepared by digesting intact VEGF-A_165_ with NE [14, 15]. Briefly, recombinant human VEGF-A_165_ (10 μg) was reconstituted in 100 μl of 44 mM sodium bicarbonate, pH 7.4 containing 2 μg of NE. Samples were allowed to incubate for 30 min at 37°C and the reaction was terminated by adding the irreversible NE inactivator diisopropyl fluorophosphate (DFP) to a final concentration of 1 μM. Samples were then dialyzed overnight using 10 kDa molecular weight cut off tubing against 20,000 volumes of PBS at 4°C to remove DFP. Intact VEGF was mock treated in parallel with the exception that NE was left out during the initial incubation. After dialysis, VEGFf and intact VEGF concentrations were measured using a quantitative ELISA kit from R&D Systems. Mock treated VEGF and VEGFf were aliquoted and stored at -80°C until use. Under these conditions >98% of intact VEGF is digested to VEGFf by NE [14, 15].

### Cell Culture

Bovine aortic endothelial (BAE) cells (passage 5–15) were maintained in low-glucose DMEM, supplemented with 10% CS, 5 mM L-glutamine, 100 U/ml penicillin G, and 100 μg/ml streptomycin sulfate. BAE cells were originally isolated from fresh aortas of 3–4 week old calfs by subjecting the luminal surface of the aorta to collagenase digestion [24]. Mouse macrophage/monocyte RAW 264.7 cells were a gift from Alfred Tamayo and were propagated in RPMI-1640 with 5 mM L-glutamine, 10% heat-inactivated FBS, 100 U/ml penicillin G, and 100 μg/ml streptomycin sulfate [25]. These cells were originally isolated from the ascites of mice bearing an Abelson leukemia virus-induced tumor [25]. FBS was inactivated by heating for 30 min in a 56°C water bath, mixing every 10 min. Neonatal rat aortic smooth muscle cells (SMCs) were isolated from neonatal Sprague-Dawley rats, ages 1–3 days by collagenase and elastase digestion of minced aortic medial tissue as described [26], and maintained in low-glucose DMEM, with 10% FBS, 5 mM L-glutamine, 100 U/ml penicillin G, and 100 μg/ml streptomycin sulfate and 1% nonessential amino acids. NIH3T3 cells were grown in high glucose DMEM, with 10% FBS, 25 mM HEPES, 5 mM L-glutamine, 100 U/ml penicillin G, and 100 μg/ml streptomycin sulfate. Embryonic EPCs were a generous gift from Antonis Hatzopoulos. These cells were originally isolated from E7.5 –E7.8 mouse embryos after trypsin/EDTA dissociation and cell plating on irradiated mouse embryonic fibroblasts followed by passaged on gelatin-coated plates as described [27]. Embryonic EPCs were grown in high glucose DMEM, with 10% heat inactivated FBS, 25 mM HEPES, 5 mM L-glutamine, 100 U/ml penicillin G, and 100 μg/ml streptomycin sulfate, 5 mM MEM non essential amino acids and 3.5 μl 2-mercaptoethanol.

### Cell Proliferation

RAW 264.7 cells were seeded at 3,000 cells/well in 12-well plates. The next day media was changed to serum free media +/- various VEGF/VEGFf concentrations and the cells kept for an additional three days at 37^°^C. Cell number was determined with CyQuant-NF-cell proliferation kit (Invitrogen, Carlsbad, CA).

### Cell Migration

Cell migration was measured using a modified Boyden Chamber technique [14]. We used 24-well Transwell^®^ Permeable Supports (Corning, NY) with migration inserts (5 or 8 μm pore size, 6.5 mm diameter). Serum starved BAE or RAW cells were plated onto the transwell inserts at a density of 100,000 cells/insert in serum-free medium, 25 mM HEPES, pH 7.5 and 0.05% Gelatin (v/v) and placed in 24-well plates containing binding buffer +/- chemoattractant (VEGF, VEGFf) at various concentrations. The assembled plate was placed at 37°C (5% CO_2_, humidified) for the duration of the migration time (2 h for BAE cells and 4 h for RAW cells). Once the migration time had finished, media from the lower and upper chambers were aspirated; migrated cells were washed once in PBS without Ca^2+^ and Mg^2+^ ions and the cells that had migrated to the other side of the membrane were fixed with 100% pre chilled methanol for 10 min. Cells were subsequently washed two times in PBS and the non migrated cells on the top side of the transwell membrane were swabbed using Q-tips. The migrated cells were then stained by incubating in 5 μg/ml propidium iodide in PBS (600 μl/well) for 10 min. Cells were subsequently washed two times in PBS and a microsurgery knife was used to cut out the transwell membrane. The membranes were placed on labeled glass slides with migrated cells facing up. A drop of Antifade Component A (Molecular Probes, Invitrogen, Carlbad, CA) was placed on top of each membrane and then covered with a glass cover slip. The coverslips were sealed to the glass slide with nailpolish. Images of migrated cells were captured by fluorescent microscopy at four to six separate non-overlapping fields/membrane at 100 x magnification. The migrated cells were counted using Image J NIH software. In experiments using EPCs, the procedure was the same as described above, with the following alterations: prior to the migration experiment EPCs were treated ± dibutyl cAMP (0.5 mM) for 48 hours in order to induce the cells to differentiate. The rest of the procedure remains as described above.

### Generation of NIH3T3 Stably Overexpressing Recombinant Human VEGF_165_

Mammalian pCIneoVEGF plasmid containing full length recombinant human VEGF_165_ cDNA was used to overexpress VEGF in NIH3T3 cells. NIH3T3 cells were transfected with pCIneoVEGF using LipofectAMINE (Invitrogen, Carlsbad, CA). Stable NIH3T3-VEGF cells were selected using 500 μg/ml G418. A heterogenic pool of transfected cells were used for experiments. VEGF overexpression was analyzed by human VEGF ELISA and quantitative RT-PCR.

### Real Time Polymerase Chain Reaction

Total RNA was extracted in 4 M guanidinium thiocyanate from cell cultures as described previously [28]. Genomic DNA was removed by incubation with RNase-free DNase I (M0303S; New England BioLabs) in the presence of RNase inhibitor. The RNA was annealed with oligo(dT) and random hexamer primers, and first-strand synthesis was carried out with MuLV reverse transcriptase. Negative controls were performed without reverse transcriptase. Real-time PCR was performed on ABI 7300 using ABI TaqMan gene expression assays. The cycling parameters were 50°C for 10 min, 95°C for 2 min, 45 cycles of 95°C for 15 sec, and 60°C for 1 min. Results were calculated using the ΔΔC_T_ method using 18S rRNA as the endogenous control [15].

### Cell Migration in Co-Culture Systems

RAW 264.7 and BAE Cell Migration in Co-culture with NIH3T3 or NIH3T3-VEGF Cells. On day 1, high or low VEGF producing NIH3T3 cells were seeded at 250,000 cells/ml in 24-well plates. On the second day, media was changed to serum free media and cells incubated for an additional day. In parallel, BAE and RAW 264.7 cells were seeded in P100 plates on day 1. On day 2 the media was changed to serum free and the cells were incubated overnight. Co-culture migration experiments were performed on day 3. Day 3: various NE concentrations were added to NIH3T3/VEGF cells and incubated at 37^°^C for 30 min. Subsequently NE was inhibited by the addition of α1-antitrypsin (α1-AT, generously provided by Dr. Phillip Stone, Department of Biochemistry, Boston University School of Medicine, Boston, MA) and cells incubated for an additional 30 min. In the mean time, RAW 264.7 or BAE cells were harvested and resuspended in serum free media (the same media that NIH3T3 cells were in) with 0.05% gelatin and 25 mM HEPES and seeded onto 5 μm pore size membrane inserts at 100,000 cells/insert. The inserts with BAE and RAW 264.7 cells were then placed on top of NIH3T3/VEGF cells +/- NE and α1-AT and the plate incubated at 37^°^C for the indicated period of time and the number of migrated cells determined as described in *Cell Migration* section.

RAW and BAE Cell Migration in Co-culture with Rat SMCs Kept under Hypoxia or Normoxia. These sets of experiments contain SMCs as the low or high VEGF producing cells. Rat SMCs were kept in culture for various periods. Three days before the co-culture migration experiment, one 24-well SMC plate was placed in a Galaxy 48R CO_2_ hypoxic incubator (Eppendorf North America, Hauppague, NY) set at 1% pO_2_ and the other plate was kept in normoxic conditions. On the second day media was changed to serum free media and the cells maintained for an additional day. Then, SMCs were treated with various NE concentrations for 30 min. NE was inhibited by the addition of α1-AT (100 μg/ml). Serum starved RAW or BAE cells were then plated on transwell inserts at 100,000 cells/insert in serum free media with 0.05% gelatin and 25 mM HEPES and placed on top of the SMC. In some instances Fc-VEGFR1/R2 chimeras (0.1 nM) were added to SMC +/- NE +/- α1-AT media for the duration of the co-culture migration to inhibit released VEGF/VEGFf.

Embryonic EPC cell migration in co-culture with rat SMCs kept under normoxia or hypoxia. Rat SMCs were kept in culture for various periods. Three days before the co-culture migration experiment, one 24-well SMC plate was placed in a hypoxic chamber (1% pO_2_) and the other plate was kept in normoxic conditions. On the second day media was changed to serum free media and the cells maintained for an additional day. Simultaneously, eEPCs were treated ± dibutyl cAMP for two days. On the day of the migration experiment, SMCs were treated with NE (0.5 μg/ml) for 30 min. NE was inhibited by the addition of α1-AT (100 μg/ml). Induced (cAMP treated) or uninduced eEPCs were then plated on transwell inserts at 100,000 cells/insert in serum free media with 1% gelatin and 25 mM HEPES and placed on top of the SMC. In some instances VEGF receptor inhibitor (0.1 nM) was added to SMC for the duration of the co-culture migration. The number of migrated cells was determined as described in the *Cell Migration* section.

### Statistical Analysis

Statistical significance of data was evaluated using one or two-way analysis of variance (ANOVA) followed by the Tukey’s multiple-comparisons test, where appropriate, using GraphPad Prism version 6.0 (San Diego, CA). Differences were considered significant when p values were < 0.05. Data are presented as the means of replicate samples from a selected experiment ± SE as indicated in the legends to figures. All results were reproduced in at least 4 separate experiments. In some repeat experiments specific conditions were sometimes altered (i.e., cell line passage number, VEGF concentration), yet all key findings were qualitatively the same as those shown in the figures. In instances where only two groups were compared, a student’s *t*-test was used to evaluate statistical significance.

## Results

### Elastase-generated VEGF Fragment Stimulates Macrophage Migration

**Fig 1:**
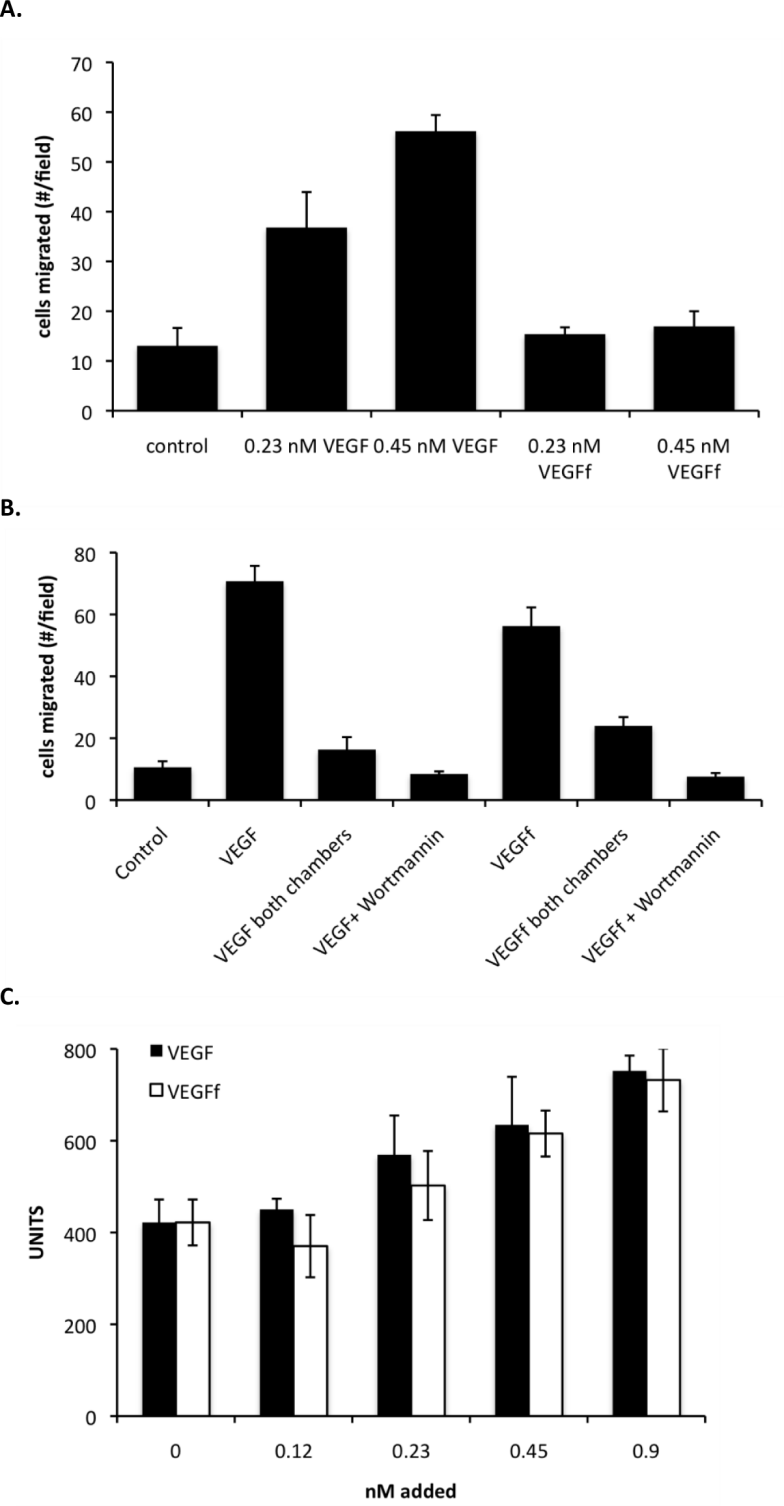
VEGFf activities on BAE and RAW 264.7 cells. *A*: BAE cells were seeded on the upper membrane of transwell cell migration chambers. VEGF or VEGFf (0.23 and 0.45 nM each) were added to the lower chamber. The cells were incubated for 2 h at 37^°^C. Migrated cells were fixed, stained with propidium iodide and images acquired with fluorescent microscope (100 x magnification). Average number of cells migrated ± SE of 12 separate fields were measured in each experiment. Similar results were observed in at least 4 separate experiments. *B*: RAW 264.7 cells were treated with or without ± 250 nM Wortmannin for 24 hr and then seeded in the upper chamber of the transwell migration apparatus (5 μm pore size). VEGF or VEGFf (0.45 nM) were added either only in the lower chamber or in the lower and upper migration chambers. The cells were incubated for 4 h at 37^°^C, and migrated cells were fixed and stained with propidium iodide and images acquired with a fluorescent microscope (100 x magnification). The number of migrated cells were counted in 12 fields per condition and presented as the average ±S.E. *C*: RAW 264.7 cells were seeded at 3000 cells/well in 12-well plates. The next day the media was changed to serum free media ± VEGF/VEGFf (0.12, 0.23, 0.45, and 0.9 nM) and the cells kept for an additional three days at 37^°^C. Cell number was determined with Cy-Quant-NF-cell proliferation kit. Each sample is the average of quadruplicate determinations ± SD. Two-way ANOVA followed by Tukey’s multiple *t* tests revealed significant differences between: control vs. 0.45 and 0.9 nM VEGF or VEGFf.

We have previously reported that NE cleaves VEGF to produce a fragmented form that has altered activity [14, 15]. Interestingly, VEGFf was able to induce Erk1/2 phosphorylation in a macrophage cell line but not in endothelial cells [15]. VEGFf stimulated Akt phosphorylation in both cell types suggesting a complex mode of regulation of VEGF through proteolytic processing [15]. Chemotaxis is a critical component of wound healing and inflammation, and VEGF has been shown to possess this activity for a variety of cell types including monocytes and endothelial cells. Therefore, we explored the chemotatic activity of VEGF and VEGFf with BAE cells and the macrophage/monocyte RAW 264.7 cell line. BAE cells exhibited increased migration in response to intact VEGF (one-way ANOVA followed by Tukey’s *t*-test, p < 0.001) but showed no response to the same concentration of VEGFf (Fig 1A). In contrast, RAW 264.7 cells showed significantly enhanced migration to both VEGF and VEGFf (Fig 1B), as determined by one-way ANOVA and Tukey’s *t*-test, p < 0.0001. The ability of each agent to stimulate RAW 264.7 cell migration was lost upon chemotactic gradient elimination by including VEGF/VEGFf in both (the top and bottom) reservoirs of the transport chamber. Since Akt activation has been demonstrated to be required for VEGF-mediated chemotaxis [29–31], the PI3-kinase inhibitor Wortmannin was included in some conditions. Neither VEGF nor VEGFf were able to stimulate RAW 264.7 cell migration in the presence of Wortmannin consistent with the previously defined role of Akt in VEGF-induced migration. As an additional evaluation of VEGF and VEGFf on RAW 264.7 cells we observed that both were able to stimulate cell proliferation to a similar degree (Fig 1C).

### Elastase Stimulates Macrophage Migration towards VEGF Producing Cells

**Fig 2:**
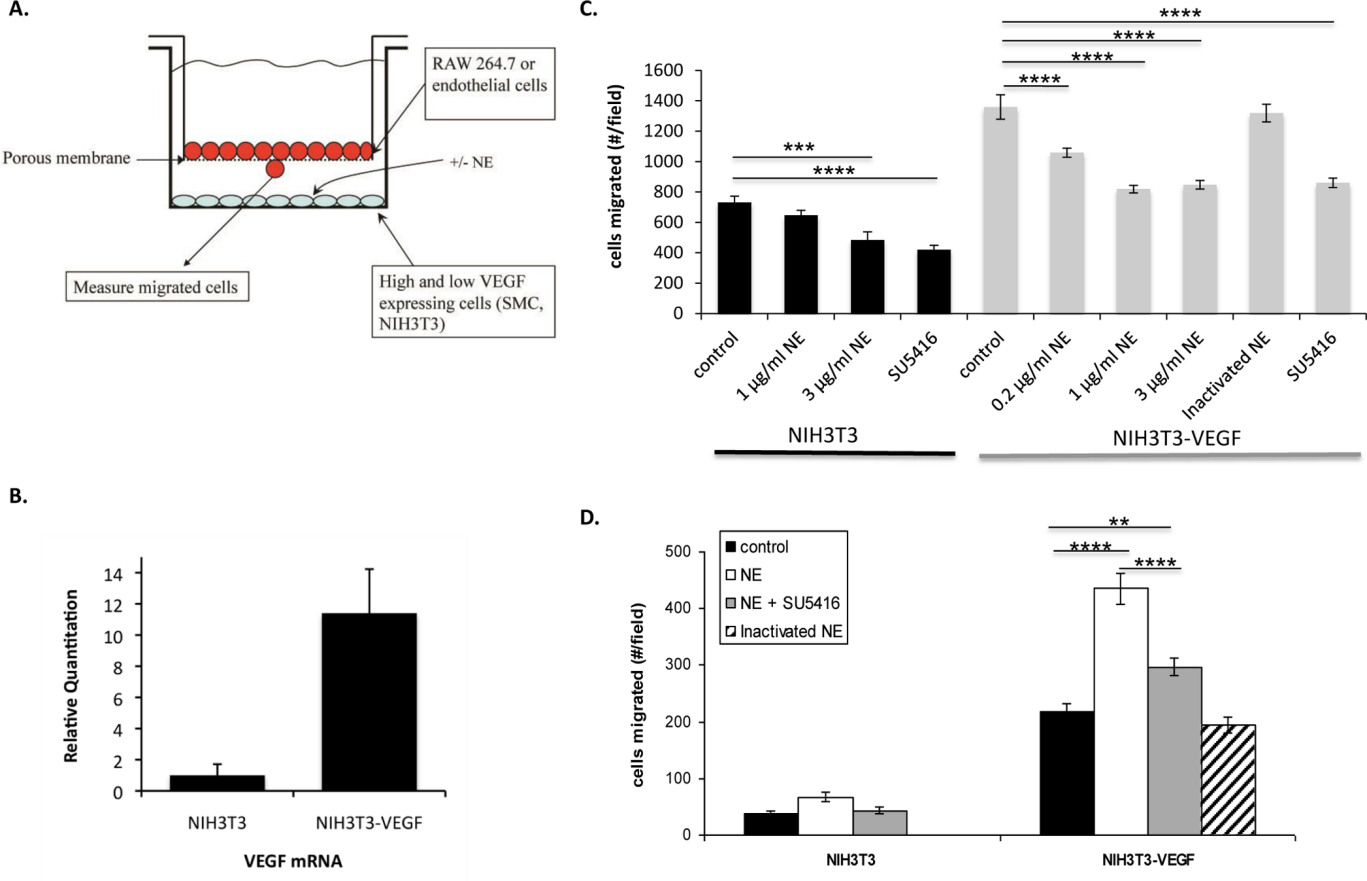
Chemotactic activities of endogenously produced VEGF/VEGFf. *A*: Schematic representation of co-culture system. *B*: Total RNA was collected from confluent NIH3T3/VEGF cells and relative real time PCR was performed to measure VEGF mRNA expression. Results were calculated using the ΔΔC_T_ method using 18S rRNA as the endogenous control. Each data point is the mean of duplicate samples each measured in triplicate ± SE. *T* test revealed significant difference between NIH3T3 and NIH3T3-VEGF (*P* < 0.001). *C*: NIH3T3 and NIH3T3-VEGF cells were grown to confluence and treated with NE for 30 minutes at 37^°^C followed by addition of α1-antitrypsin directly to the media. BAE cells ± SU5416 (10 μM) were plated in the top chamber of the transwells (5 μm pore size) and allowed to migrate for 2 h. Migrated cells were fixed, stained with propidium iodide and images acquired with a fluorescent microscope (100 x magnification). The average number of migrated cells ± SE was determined in 12 fields per condition. Two-way ANOVA followed by Tukey’s test was performed. *** = p < 0.001; **** = p < 0.0001. *D*: NIH3T3 and NIH3T3-VEGF cells were grown to confluence in the lower chamber of transwells and treated with NE for 30 min at 37^°^C followed by addition of α1-antitrypsin directly to the media. RAW 264.7 cells ± SU5416 (10 μM) were plated in the top chamber of the transwells (5 μm pore size) and allowed to migrate for 3.5 h. Migrated cells were fixed, stained with propidium iodide and images acquired with a fluorescent microscope (100 x magnification). The average number of migrated cells ± SE was determined in 12 fields per condition. Two-way ANOVA followed by Tukey’s test was performed. ** = p < 0.01; **** = p < 0.0001.

The release of NE at sites of inflammation may modulate VEGF bioavailability by degrading VEGF binding sites in the ECM causing release of VEGF, and may also alter VEGF activity by directly cleaving VEGF. Hence, the response to NE-induced damage may be dependent on the specific target cell’s responsiveness to VEGF and VEGFf. In order to test these possibilities, we established a co-culture model system using NIH3T3 cells that were engineered to express high levels of VEGF as a source of cell/ECM derived VEGF (Fig 2A). To verify VEGF synthesis in the engineered NIH3T3 cells we conducted ELISA and qPCR analysis. There was no detectable recombinant human VEGF in the control NIH3T3 cell lysates or media while there was 195±31 pg/ml VEGF in the cell lysates and 694±71 pg/ml VEGF in the media from the VEGF transfected cells (NIH3T3-VEGF). Similarly, qPCR showed ~11-fold greater VEGF mRNA levels in NIH3T3-VEGF cells compared to vector transfected control NIH3T3 cells (Fig 2B). The NIH3T3 and NIH3T3-VEGF cells were cultured on the bottom surface of the migration chamber and were subjected to mild NE treatment. After inactivation of the NE with α1-antitrypsin, BAE or RAW 264.7 cells were plated into the upper chamber of the apparatus and migration was evaluated.

BAE cells showed increased migration towards the NIH3T3-VEGF cells compared to the control NIH3T3 cells. Interestingly, the addition of NE reduced migration of BAE cells in co-culture with NIH3T3 and NIH3T3-VEGF cells. A similar decrease in BAE cell migration was mimicked by the addition of a VEGF receptor tyrosine kinase inhibitor, SU5416, indicating that a significant portion of the migratory response was due to VEGF produced by NIH3T3-VEGF cells and that digestion of intact VEGF to VEGFf by NE is likely responsible for the reduction in BAE cell migration after NE treatment (Fig 2C). As an important control, we confirmed that BAE cell migration was not altered by the presence of pre-inactivated NE or contaminants present in the NE or α1-antitrypsin preparations.

In contrast to BAE cell response, NE enhanced migration of RAW 264.7 cells in co-culture with VEGF producing cells. The increased activity was related to VEGF/VEGFf released by NE, as addition of the VEGF receptor kinase inhibitor, SU5416, attenuated this response to a large degree. It is also apparent that the number of RAW cells migrating towards NIH3T3-VEGF cells in each condition was higher than towards low VEGF producing cells. However, it is important to note that NE stimulated a small but significant increase in RAW migration when in co-culture with control (non-VEGF expressing) NIH3T3 cells (Fig 2D). A portion of this response was also related to VEGF/VEGFf released, as shown by the reduction in migration upon VEGFR inhibition, which likely reflects the action of low levels of endogenous VEGF expressed by NIH3T3 cells. As with BAE cells, the effects of NE were not due to side effects of NE or α1-antitrypsin presence, as pre inactivated NE did not alter RAW 264.7 cell migration.

### Elastase Stimulates Macrophage Migration to Hypoxic Vascular Smooth Muscle Cells

**Fig 3:**
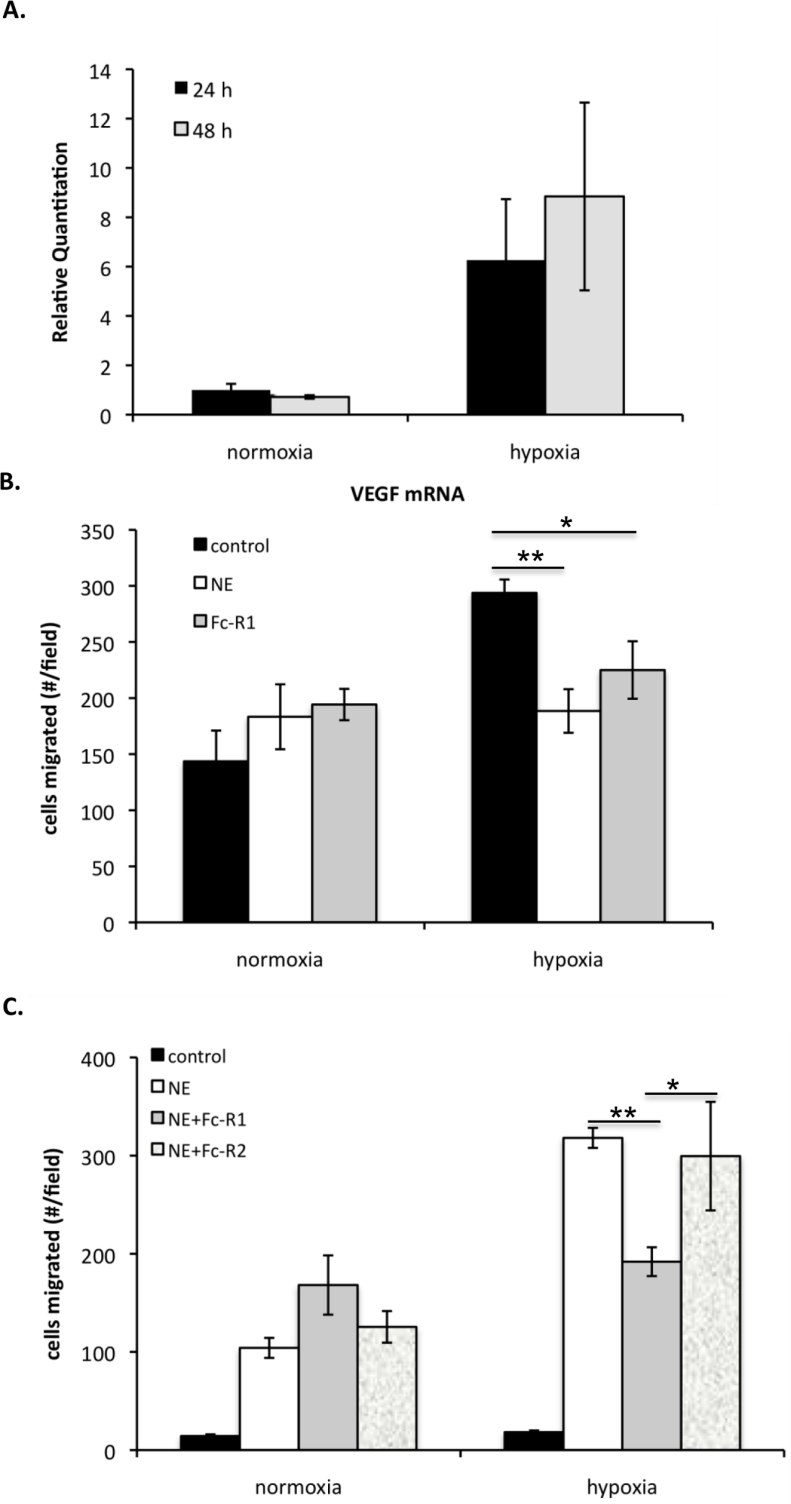
Chemotactic activities of hypoxia-induced VEGF and NE-generated VEGFf. *A*: SMCs were kept in normoxia or hypoxia (1% pO_2_) for 24 and 48 hours. Total RNA was collected and relative real time PCR was performed to measure VEGF mRNA expression. Results were calculated using the ΔΔC_T_ method using 18S rRNA as the endogenous control. Each data point is the mean of duplicate samples each measured three times ± SE. *T* test revealed significant difference between normoxia and hypoxia (*P* < 0.001). *B*: SMCs were placed in hypoxia (1% pO_2_) or normoxia for 48 h then treated ± 3 μg/ml NE for 30 minutes and α1-antitrypsin was added directly to the media ± Fc-VEGFR1 chimera (100 ng/ml). BAE cellss were placed into transwells and allowed to migrate for 3.5 h. Migrated cells were fixed, stained with propidium iodide and images acquired with a fluorescent microscope (100 x magnification). The average number of migrated cells ± SE was determined in 12 fields per condition. Two-way ANOVA followed by Tukey’s was performed. * = p < 0.05; ** = p < 0.01. *C*: SMCs were placed in hypoxia (1% pO_2_) or normoxia for 48 h then treated ± 5 μg/ml NE for 30 minutes and α1-antitrypsin was added directly to the media ± Fc-VEGFR1/2 chimeras (100 ng/ml). RAW264.7 cells were placed into transwells and allowed to migrate for 3.5 h. Migrated cells were fixed, stained with propidium iodide and images acquired with a fluorescent microscope (100 x magnification). The average number of migrated cells ± SE was determined in 12 fields per condition. Two-way ANOVA followed by Tukey’s was performed. There was a significant difference between control and each treatment arm under normoxia and hypoxia that is not indicated in the graph. ** = p < 0.01; * = p < 0.05.

VEGF mRNA expression is induced by exposure to low oxygen tension under a variety of pathophysiological conditions [32]. Thus, we noted that rat aortic smooth muscle cells (SMCs) can be induced to produce high levels of VEGF when grown under hypoxic conditions (1% pO_2_). Quantitative ELISA showed that SMCs placed in hypoxia for 48 hours exhibited a dramatic (10–100 fold) increase in VEGF protein levels (not shown). Moreover, relative RT-PCR showed an increase in VEGF mRNA 24 and 48 h after SMCs were subjected to hypoxia compared to normoxic conditions (Fig 3A). Thus, this cell culture system provides a means to modulate VEGF levels in a manner that is characteristic of tissue injury.

We wanted to evaluate whether NE treatment of SMCs grown in hypoxia would modulate BAE cell migration and whether this response is due to VEGF/VEGFf being released. BAE cells showed increased migration toward SMCs that had been subjected to hypoxia compared to control SMCs, and as expected, this migratory response towards hypoxic SMCs was reduced by NE digestion (Fig 3B). To delineate what fraction of this migratory response was due to VEGF/VEGFf release by NE we took advantage of soluble Fc-VEGFR1/2 chimeras. Fc-VEGFR1 chimera can bind both intact and cleaved VEGF to inhibit receptor activation on cells [14, 15]. Addition of Fc-VEGFR1 chimera to the shared media resulted in a significant drop in BAE cell migration when in co-culture with SMCs grown in hypoxia and caused no change when added to co-cultures maintained under normoxic conditions. Thus, BAE cell migration towards hypoxic SMCs appears, in part, dependent on VEGF, and this response is likely attenuated by NE via the generation of VEGFf.

RAW cell migration in co-culture with SMCs was evaluated. NE treatment enhanced RAW cell migration in co-culture with SMCs grown under both, normoxia and hypoxia, with the response being greater with the cells that had been exposed to hypoxia (Fig 3C). To delineate the fraction of this migratory response that was due to VEGF/VEGFf release by NE we took advantage of soluble Fc-VEGFR1/2 chimeras. Based on our previous study, the Fc-VEGFR1 chimera is able to bind VEGF and VEGFf whereas Fc-VEGFR2 is only able to bind VEGF but not VEGFf. Thus, these reagents can be used to distinguish between the role of VEGF and VEGFf. NE-stimulated RAW cell migration to hypoxic SMCs was reduced by the Fc-VEGFR1 chimera while the same chimera had no effect on RAW cell migration with normoxic SMCs. Thus, a fraction of the migratory response to hypoxic SMCs is likely due to VEGFf release, whereas the NE-induced migration of RAW cells with normoxic SMCs appears to involve factors other than VEGF. Addition of Fc-VEGFR2 along with NE did not reduced NE-stimulated RAW cell migration with hypoxic or normoxic SMCs, suggesting that there is little intact VEGF released. These results indicate that RAW cells migrate towards a NE-generated VEGFf released from hypoxic SMCs.

### NE-generated VEGFf acts as a chemotactic factor for endothelial progenitor cells

The identification of putative bone marrow-derived circulating endothelial progenitor cells from human peripheral blood challenged the notion of angiogenesis as the exclusive mechanism responsible for new blood vessel formation in postnatal life and re-introduced the concept whereby progenitor cells differentiate into mature endothelial cells and contribute to postnatal neovascularization [33–35]. While there remains dispute over the relative role of EPCs in angiogenesis, this is a topic of active research [36, 37]. Assessing EPC activity is important in light of the possibility that these cells can home in on injured areas and differentiate into endothelial cells, assisting in vascular repair and in the formation of new blood capillaries. However, the response to vascular injury is a complex process involving a series of events such as the recruitment of inflammatory cells, changes to SMC phenotype and ECM production, EPC recruitment and maturation, and endothelial repair.

**Fig 4:**
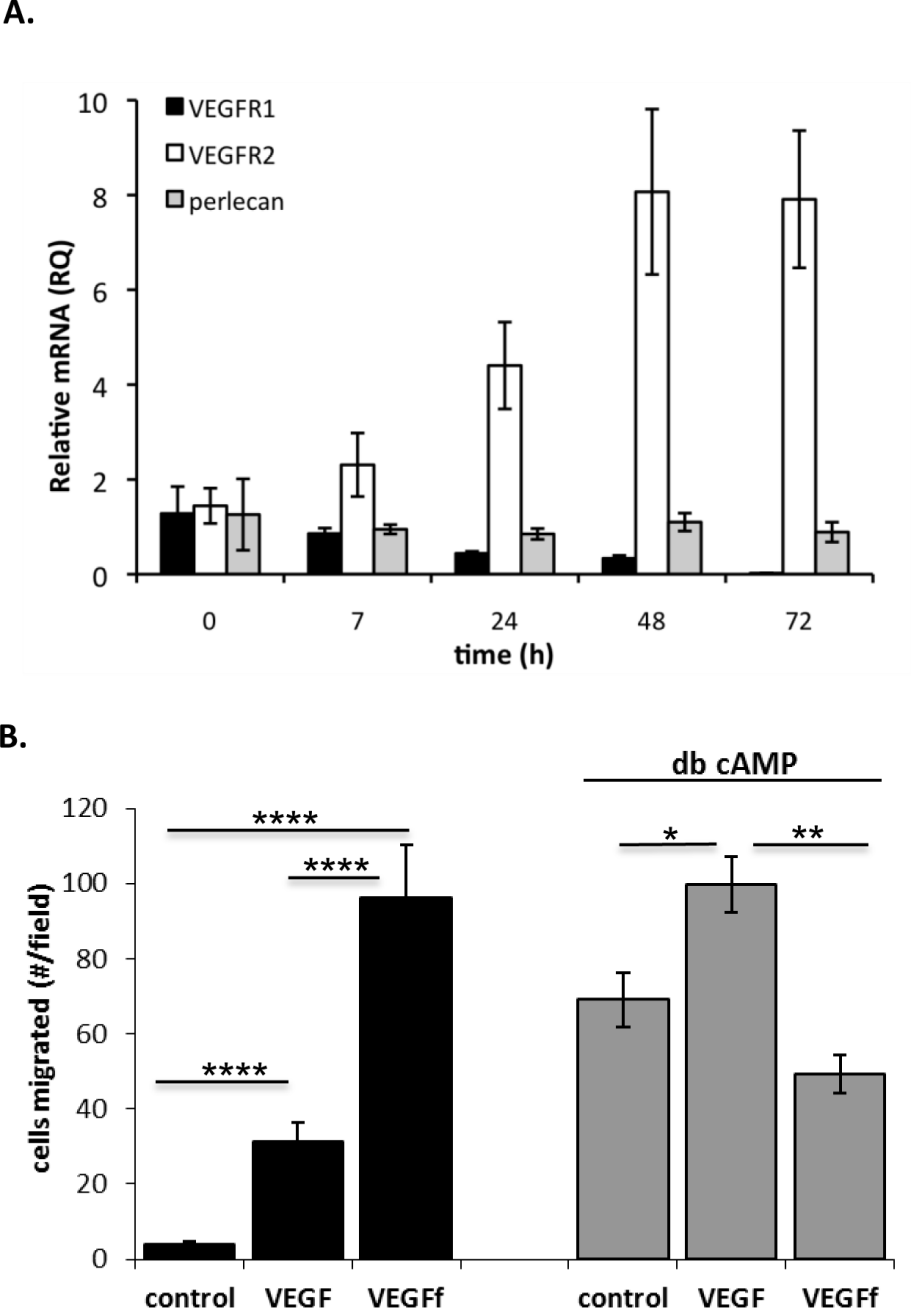
VEGFf effect on eEPC migration. *A*: Total RNA was collected from confluent eEPCs ± db cAMP and relative real time PCR was performed to measure VEGFR1, VEGFR2 and perlecan mRNA expression. Results were calculated using the ΔΔC_T_ method using 18S rRNA as the endogenous control. *B*: eEPC ± db cAMP were plated into transwell chambers (8 μm pore size) and VEGF or VEGFf (0.23 nM) were included in the lower chamber. Cells were allowed to migrate for 6 h; migrated cells were fixed, stained with propidium iodide, and images acquired with a fluorescent microscope (100 x magnification). The average number of migrated cells ± SE was determined in 12 fields per condition. Two-way ANOVA followed by Tukey’s was performed. * = p < 0.05; ** = p < 0.01; **** = p < 0.0001.

In order to examine VEGFf activities on progenitor cell migration, we used a stable mouse embryonic endothelial progenitor cell (eEPC) line [27, 38, 39]. This cell line is useful as it can be reproducibly induced to undergo endothelial differentiation by the addition of dibutyl cAMP. Under these conditions a dramatic shift in VEGF receptor expression, as indicated by a reduction in VEGFR1 and increase in VEGFR2 mRNA levels, was noted as the cells mature from progenitor to endothelial cells (Fig 4A). Perlecan, a major proteoglycan produced by a variety of cells including endothelial cells, was used as a control, and the levels remained unchanged. Thus, we investigated if responsiveness to VEGF and VEGFf varied as these cells differentiate. Embryonic EPCs were induced to differentiate with dibutyl cAMP or maintained in the progenitor state for 48 h prior to stimulation with VEGF and VEGFf. Uninduced eEPCs showed enhanced migration in response to VEGFf compared to VEGF. However, when these cells were induced to mature with dibutyl cAMP the response to VEGFf was selectively eliminated and the cells responded in a manner that was qualitatively similar to that of endothelial cells (Fig 4B).

**Fig 5:**
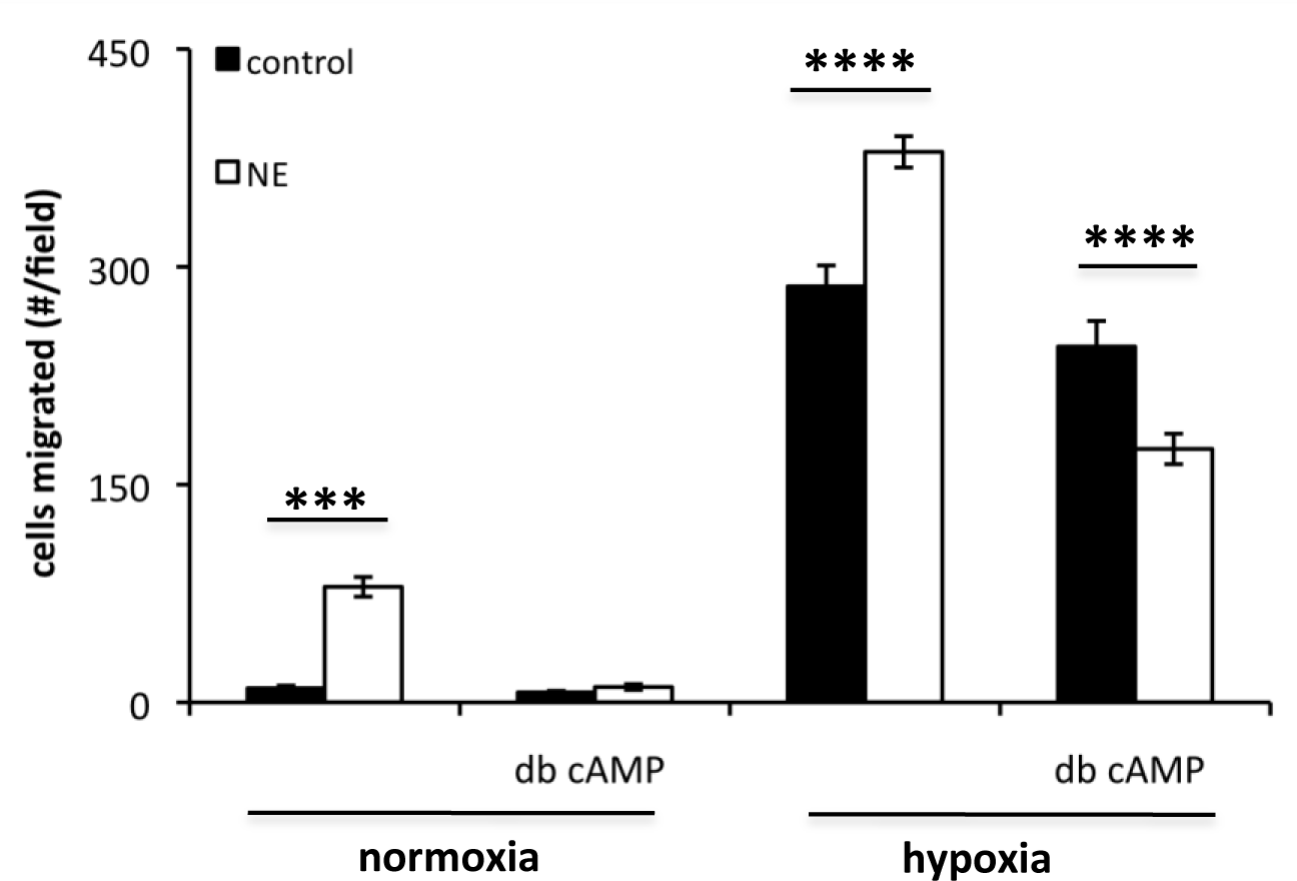
eEPC migration in co-culture with SMC ± hypoxia. SMCs were placed in hypoxia (1% pO_2_) or normoxia for 48 h then treated ± 0.5 μg/ml NE for 30 minutes and α1-antitrypsin was added to inhibit NE. eEPCs (± db cAMP for 48 h) were placed into transwells (8 μm) and allowed to migrate for 3 h. Migrated cells were fixed, stained with propidium iodide and images acquired with a fluorescent microscope (100 x magnification). The average number of migrated cells ± SE was determined in 12 fields per condition; *** = p < 0.001; **** = p < 0.0001.

To determine whether this migratory response is indicative of an endogenous process whereby VEGF-laden cells/ECM can provide eEPC stimulation, we performed co-culture experiments using SMCs as the VEGF source. SMCs were subjected to 48 h hypoxia or normoxia pretreatment prior to conducting eEPC migration experiments. Before eEPC addition to the co-cultures, SMCs were subjected to NE pretreatment for 30 min in order to release VEGF/VEGFf from the SMC-ECM. As can be observed in Fig 5, NE stimulated the migration of uninduced eEPCs, and this activity was more pronounced with hypoxic SMCs. This effect was significantly reduced by inhibition of VEGF receptors on eEPCs with SU5416 (data not shown). As predicted from the experiments described above with purified VEGF/VEGFf, the stimulatory effect of NE was attenuated in eEPCs that were induced to differentiate with dibutyl cAMP.

## Discussion

In the present study we examined the migratory response of BAE cells, RAW 264.7 macrophages/monocytes as well as embryonic endothelial progenitor cells in co-culture with high and low VEGF producing cells with or without NE treatment. We observed that NE stimulated RAW 264.7 macrophage migration most likely by generating VEGFf from NIH3T3 cell matrices. Interestingly, BAE cells migrated towards high VEGF producing cells when in the absence of NE, and this response was mediated by VEGF. NE treatment reduced BAE cell migration, likely due to VEGF degradation to VEGFf, to which BAE cells do not respond. Similar results were obtained using co-cultures of RAW 264.7 or BAE cells with SMCs subjected to hypoxia treatment. Finally, we showed that endothelial progenitor cells migrated towards SMCs pre-conditioned in hypoxia, and the effect was increased with NE treatment. The migratory effect was reduced upon stimulation of eEPC maturation to endothelial-like cells. We were able to simulate proteolytic tissue injury in this co-culture system and explore the potential contribution of ECM-resident growth factors to the recruitment of various cells.

It is established that angiogenesis and neovascularization are both necessary for normal vascular tissue function and repair after injury. However, one of the first steps in blood vessel growth is the degradation of the sub endothelial basement membrane and the surrounding ECM. Therefore, proteolytic degradation is an integral part of neovascularization. Importantly, proteinases can switch on angiogenesis by liberating matrix-bound angiogenic activators and proteolytically activating angiogenic chemokines [40–42]. Bearing in mind the critical role of the ECM in vessel growth and maintenance, it is understandable that proteolytic remodeling of the ECM must occur in a controlled fashion. Here we show evidence that proteolytic injury by NE might contribute to the recruitment of critical cell types involved in vascular tissue repair. As such, the extent of the proteolytic injury would be predicted to lead to either endothelial tissue repair (e.g. intact VEGF release from ECM that can contribute to endothelial cell migration and survival as well as EPC recruitment) or to the propagation of the inflammatory response (monocyte recruitment) and excessive proteolytic tissue degradation.

Inflamed or injured tissue is often hypoxic and can induce angiogenesis through upregulation of factors such as VEGF among other regulators of angiogenesis. The regulation of angiogenesis and endothelial repair is probably crucial to proper healing, although it remains poorly understood. In our co-culture studies, SMCs grown under hypoxic conditions showed a dramatic increase in VEGF expression. Exposure of SMCs to hypoxia led to enhanced migration of co-cultured endothelial cells. Treating SMCs with NE resulted in increased migration of co-cultured macrophages, but a drop in endothelial cell migration. These results support the notion that VEGF is an important mediator of vascular remodeling in inflamed tissues, contributing to both endothelial cell migration and proliferation, and the propagation of the inflammatory response.

Studies have reported the presence of endothelial progenitor cells in the endothelial surfaces and the intimal regions of pulmonary arteries of patients with COPD [43]. Interestingly, the number of progenitor cells was associated with the response to hypoxic stimulus and with the enlargement of the arterial wall. Consequently, we were able to show that endothelial progenitor cells were able to migrate towards hypoxic cells (co-culture experiments with SMCs grown in hypoxia). Neutrophil elastase treatment of SMCs pre-conditioned in hypoxia led to additional increase in progenitor cell migration. Taken together, these studies suggest that the progenitor cells might be recruited to injured and hypoxic tissue where they may contribute to endothelial repair and vessel remodeling through VEGF-related signals.

**Fig 6:**
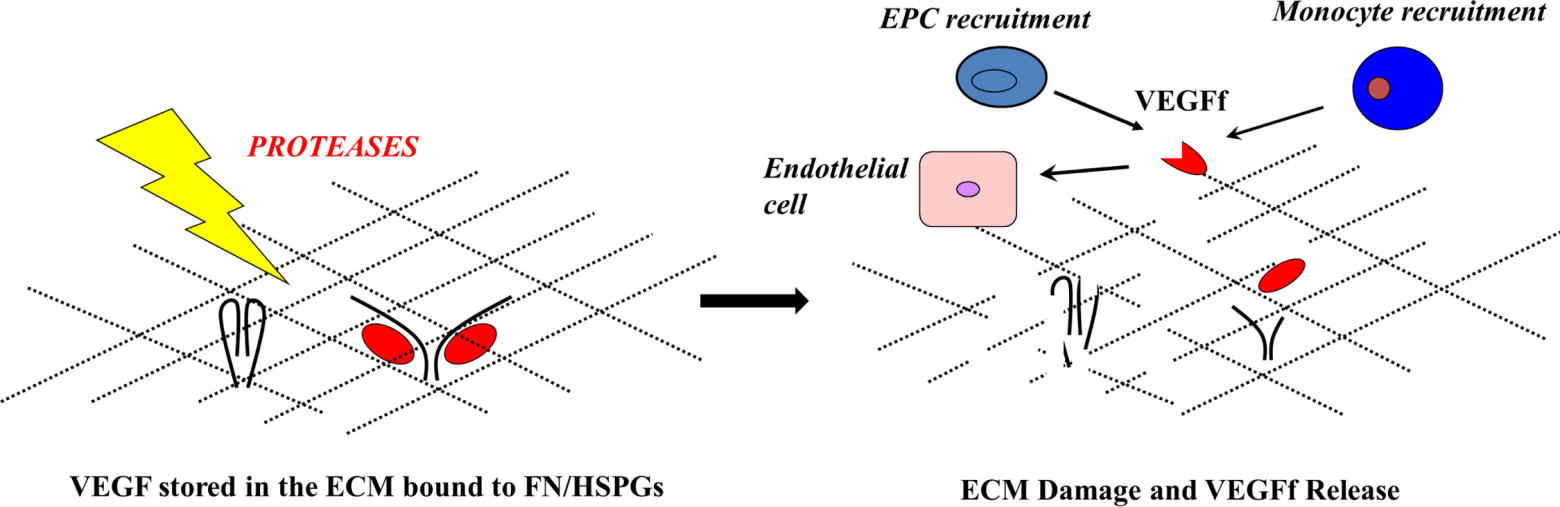
Schematic representation of elastase-mediated VEGFf generation, release and recruitment of circulating cells to the injured tissue. VEGF is stored in the extracellular matrix (ECM). Neutrophils are the first innate immune cells to be recruited to the site of injury and secrete proteases (e.g. neutrophil elastase). Neutrophil elastase is able to modulate VEGF dynamics within ECM by degrading fibronectin (FN) and heparan sulfate proteoglycan (HSPGs) binding sites in the ECM as well as by directly cleaving VEGF and releasing VEGFf to act on surrounding cells [14, 15]. The extent of proteolytic injury would dictate the ratio of intact and cleaved VEGF being released. As such, endothelial tissue repair would be promoted through VEGF/VEGFf acting on neighboring endothelial cells and circulating endothelial progenitor cells, promoting neo/angiogenesis. In the event of extensive proteolytic injury, the majority of released VEGF might be the more diffusible VEGFf, preferentially binding to VEGFR1 on macrophage/monocytic cells, leading to their recruitment, propagation of the inflammatory response and excessive proteolytic tissue degradation. This schematic representation is simplified and is not intended to exclude the possibility that other proteases (e.g., MMPs) may also influence VEGF release during tissue injury and inflammation.

In this study, we report that NE-mediated digestion of ECM appears to release both fragmented and intact VEGF through the action of NE directly on VEGF and indirectly on VEGF-binding sites (e.g., heparan sulfate proteoglycans [44, 45]). It is possible that, under certain conditions, VEGFf may function to enhance the activity of VEGF on endothelial cells by occupying decoy VEGFR1 on endothelial cells thus enhancing the association of intact VEGF with VEGFR2. Indeed, we have previously found that the presence of VEGFf can enhance the activity of intact VEGF on endothelial cells when present at the proper ratio [14]. Under the conditions used in this study we did not observe enhancing activity of VEGFf, likely because the NE concentrations used were high enough to significantly reduce intact VEGF levels. Since VEGFf does not bind to the major VEGF-binding sites in the ECM [14], release of VEGFf would be expected to lead to enhance diffusion to distant sites where it might recruit macrophages and progenitor cells to the site of injury (Fig 6) [14]. However, chronic or excessive elastolysis might lead to complete conversion of VEGF to VEGFf, with the concomitant loss of critical endothelial activities and consequently, excessive inflammatory cell activation. A more complete understanding of the role of VEGF within proteolytically injured regions will need to consider the possible function of proteolytic fragments of VEGF.

## References

[1] Gooptu B, Ekeowa UI, Lomas DA. Mechanisms of emphysema in alpha1-antitrypsin deficiency: molecular and cellular insights. The European respiratory journal: official journal of the European Society for Clinical Respiratory Physiology. 2009;34: 475–88. 10.1183/09031936.00096508 19648523

[2] Tuder RM, Yoshida T, Fijalkowka I, Biswal S, Petrache I. Role of lung maintenance program in the heterogeneity of lung destruction in emphysema. Proc Am Thorac Soc. 2006;3: 673–9. 17065372 10.1513/pats.200605-124SF PMC2647653

[3] Winkler T, Suki B. Emergent structure-function relations in emphysema and asthma. Crit Rev Biomed Eng. 2011;39: 263–80. 22011233 10.1615/critrevbiomedeng.v39.i4.20 PMC3228247

[4] Barnes PJ, Shapiro SD, Pauwels RA. Chronic obstructive pulmonary disease: molecular and cellular mechanisms. Eur Respir J. 2003;22: 672–88. 14582923 10.1183/09031936.03.00040703

[5] Shapiro SD. Proteolysis in the lung. Eur Respir J Suppl. 2003;44: 30s–2s. 14582898 10.1183/09031936.03.00000903a

[6] Tuder RM, Yun JH. Vascular endothelial growth factor of the lung: friend or foe. Curr Opin Pharmacol. 2008;8: 255–60. 10.1016/j.coph.2008.03.003 18468486 PMC2622735

[7] Voelkel NF, Vandivier RW, Tuder RM. Vascular endothelial growth factor in the lung. Am J Physiol Lung Cell Mol Physiol. 2006;290: L209–21. 16403941 10.1152/ajplung.00185.2005

[8] Barbera JA, Peinado VI. Disruption of the lung structure maintenance programme: a comprehensive view of emphysema development. The European respiratory journal: official journal of the European Society for Clinical Respiratory Physiology. 2011;37: 752–4. 10.1183/09031936.00154610 21454894

[9] Peinado VI, Pizarro S, Barbera JA. Pulmonary vascular involvement in COPD. Chest. 2008;134: 808–14. 10.1378/chest.08-0820 18842913

[10] Kasahara Y, Tuder RM, Cool CD, Lynch DA, Flores SC, Voelkel NF. Endothelial cell death and decreased expression of vascular endothelial growth factor and vascular endothelial growth factor receptor 2 in emphysema. Am J Respir Crit Care Med. 2001;163: 737–44. 11254533 10.1164/ajrccm.163.3.2002117

[11] Kasahara Y, Tuder RM, Taraseviciene-Stewart L, Le Cras TD, Abman S, Hirth PK, et al Inhibition of VEGF receptors causes lung cell apoptosis and emphysema. J Clin Invest. 2000;106: 1311–9. 11104784 10.1172/JCI10259 PMC387249

[12] Tang K, Rossiter HB, Wagner PD, Breen EC. Lung-targeted VEGF inactivation leads to an emphysema phenotype in mice. J Appl Physiol. 2004;97: 1559–66; discussion 49. 15208295 10.1152/japplphysiol.00221.2004

[13] Demedts IK, Demoor T, Bracke KR, Joos GF, Brusselle GG. Role of apoptosis in the pathogenesis of COPD and pulmonary emphysema. Respir Res. 2006;7: 53 16571143 10.1186/1465-9921-7-53 PMC1501017

[14] Forsten-Williams K, Kurtagic E, Nugent MA. Complex receptor-ligand dynamics control the response of the VEGF system to protease injury. BMC Syst Biol. 2011;5: 170 10.1186/1752-0509-5-170 22014244 PMC3253741

[15] Kurtagic E, Jedrychowski MP, Nugent MA. Neutrophil elastase cleaves VEGF to generate a VEGF fragment with altered activity. Am J Physiol Lung Cell Mol Physiol. 2009;296: L534–46. 10.1152/ajplung.90505.2008 19136576 PMC2660218

[16] Inoue M, Itoh H, Ueda M, Naruko T, Kojima A, Komatsu R, et al Vascular endothelial growth factor (VEGF) expression in human coronary atherosclerotic lesions: possible pathophysiological significance of VEGF in progression of atherosclerosis. Circulation. 1998;98: 2108–16. 9815864 10.1161/01.cir.98.20.2108

[17] Kalka C, Tehrani H, Laudenberg B, Vale PR, Isner JM, Asahara T, et al VEGF gene transfer mobilizes endothelial progenitor cells in patients with inoperable coronary disease. Ann Thorac Surg. 2000;70: 829–34. 11016318 10.1016/s0003-4975(00)01633-7

[18] Belgore F, Blann A, Neil D, Ahmed AS, Lip GY. Localisation of members of the vascular endothelial growth factor (VEGF) family and their receptors in human atherosclerotic arteries. J Clin Pathol. 2004;57: 266–72. 14990597 10.1136/jcp.2003.012419 PMC1770244

[19] Gillis S, Furie BC, Furie B. Interactions of neutrophils and coagulation proteins. Seminars in hematology. 1997;34: 336–42. 9347584

[20] Dollery CM, Owen CA, Sukhova GK, Krettek A, Shapiro SD, Libby P. Neutrophil elastase in human atherosclerotic plaques: production by macrophages. Circulation. 2003;107: 2829–36. 12771009 10.1161/01.CIR.0000072792.65250.4A

[21] Marino F, Tozzi M, Schembri L, Ferraro S, Tarallo A, Scanzano A, et al Production of IL-8, VEGF and elastase by circulating and intraplaque neutrophils in patients with carotid atherosclerosis. PloS one. 2015;10: e0124565 10.1371/journal.pone.0124565 25893670 PMC4404350

[22] Holmes DI, Zachary I. The vascular endothelial growth factor (VEGF) family: angiogenic factors in health and disease. Genome biology. 2005;6: 209 15693956 10.1186/gb-2005-6-2-209 PMC551528

[23] Robinson CJ, Stringer SE. The splice variants of vascular endothelial growth factor (VEGF) and their receptors. J Cell Sci. 2001;114: 853–65. 11181169 10.1242/jcs.114.5.853

[24] Dinbergs ID, Brown L, Edelman ER. Cellular response to transforming growth factor-beta1 and basic fibroblast growth factor depends on release kinetics and extracellular matrix interactions. The Journal of biological chemistry. 1996;271: 29822–9. 8939921 10.1074/jbc.271.47.29822

[25] Raschke WC, Baird S, Ralph P, Nakoinz I. Functional macrophage cell lines transformed by Abelson leukemia virus. Cell. 1978;15: 261–7. 212198 10.1016/0092-8674(78)90101-0

[26] Faris B, Tan OT, Toselli P, Franzblau C. Long-term neonatal rat aortic smooth muscle cell cultures: a model for the tunica media of a blood vessel. Matrix. 1992;12: 185–8. 1406452 10.1016/s0934-8832(11)80060-0

[27] Hatzopoulos AK, Folkman J, Vasile E, Eiselen GK, Rosenberg RD. Isolation and characterization of endothelial progenitor cells from mouse embryos. Development. 1998;125: 1457–68. 9502726 10.1242/dev.125.8.1457

[28] Wolfe BL, Rich CB, Goud HD, Terpstra AJ, Bashir M, Rosenbloom J, et al Insulin-like growth factor-I regulates transcription of the elastin gene. J Biol Chem. 1993;268: 12418–26. 8509381

[29] Ackah E, Yu J, Zoellner S, Iwakiri Y, Skurk C, Shibata R, et al Akt1/protein kinase Balpha is critical for ischemic and VEGF-mediated angiogenesis. The Journal of clinical investigation. 2005;115: 2119–27. 16075056 10.1172/JCI24726 PMC1180542

[30] Shiojima I, Walsh K. Role of Akt signaling in vascular homeostasis and angiogenesis. Circulation research. 2002;90: 1243–50. 12089061 10.1161/01.res.0000022200.71892.9f

[31] Somanath PR, Razorenova OV, Chen J, Byzova TV. Akt1 in endothelial cell and angiogenesis. Cell cycle. 2006;5: 512–8. 16552185 10.4161/cc.5.5.2538 PMC1569947

[32] Dor Y, Porat R, Keshet E. Vascular endothelial growth factor and vascular adjustments to perturbations in oxygen homeostasis. American journal of physiology Cell physiology. 2001;280: C1367–74. 11350731 10.1152/ajpcell.2001.280.6.C1367

[33] Asahara T, Murohara T, Sullivan A, Silver M, van der Zee R, Li T, et al Isolation of putative progenitor endothelial cells for angiogenesis. Science. 1997;275: 964–7. 9020076 10.1126/science.275.5302.964

[34] Risau W, Sariola H, Zerwes HG, Sasse J, Ekblom P, Kemler R, et al Vasculogenesis and angiogenesis in embryonic-stem-cell-derived embryoid bodies. Development. 1988;102: 471–8. 2460305 10.1242/dev.102.3.471

[35] Madonna R, De Caterina R. Circulating endothelial progenitor cells: Do they live up to their name? Vascular pharmacology. 2015;67-69C: 2–5. 10.1016/j.vph.2015.02.018 25869520

[36] Pearson JD. Endothelial progenitor cells—an evolving story. Microvasc Res. 2010;79: 162–8. 10.1016/j.mvr.2009.12.004 20043930

[37] Mobius-Winkler S, Hollriegel R, Schuler G, Adams V. Endothelial progenitor cells: implications for cardiovascular disease. Cytometry A. 2009;75: 25–37. 10.1002/cyto.a.20669 19009636

[38] Kupatt C, Horstkotte J, Vlastos GA, Pfosser A, Lebherz C, Semisch M, et al Embryonic endothelial progenitor cells expressing a broad range of proangiogenic and remodeling factors enhance vascularization and tissue recovery in acute and chronic ischemia. Faseb J. 2005;19: 1576–8. 16009705 10.1096/fj.04-3282fje

[39] Wei J, Blum S, Unger M, Jarmy G, Lamparter M, Geishauser A, et al Embryonic endothelial progenitor cells armed with a suicide gene target hypoxic lung metastases after intravenous delivery. Cancer cell. 2004;5: 477–88. 15144955 10.1016/s1535-6108(04)00116-3

[40] Handsley MM, Edwards DR. Metalloproteinases and their inhibitors in tumor angiogenesis. Int J Cancer. 2005;115: 849–60. 15729716 10.1002/ijc.20945

[41] Foley CJ, Fanjul-Fernandez M, Bohm A, Nguyen N, Agarwal A, Austin K, et al Matrix metalloprotease 1a deficiency suppresses tumor growth and angiogenesis. Oncogene. 2014;33: 2264–72. 10.1038/onc.2013.157 23708660 PMC4221191

[42] Fu Y, Nagy JA, Brown LF, Shih SC, Johnson PY, Chan CK, et al Proteolytic cleavage of versican and involvement of ADAMTS-1 in VEGF-A/VPF-induced pathological angiogenesis. The journal of histochemistry and cytochemistry: official journal of the Histochemistry Society. 2011;59: 463–73. 21411713 10.1369/0022155411401748 PMC3201172

[43] Peinado VI, Ramirez J, Roca J, Rodriguez-Roisin R, Barbera JA. Identification of vascular progenitor cells in pulmonary arteries of patients with chronic obstructive pulmonary disease. American journal of respiratory cell and molecular biology. 2006;34: 257–63. 16239642 10.1165/rcmb.2005-0255OC

[44] Buczek-Thomas JA, Chu CL, Rich CB, Stone PJ, Foster JA, Nugent MA. Heparan sulfate depletion within pulmonary fibroblasts: implications for elastogenesis and repair. J Cell Physiol. 2002;192: 294–303. 12124775 10.1002/jcp.10135

[45] Buczek-Thomas JA, Nugent MA. Elastase-mediated release of heparan sulfate proteoglycans from pulmonary fibroblast cultures. A mechanism for basic fibroblast growth factor (bFGF) release and attenuation of bfgf binding following elastase-induced injury. J Biol Chem. 1999;274: 25167–72. 10455199 10.1074/jbc.274.35.25167

